# Prevalence and incidence of idiopathic subglottic stenosis in southern and central Alberta: a retrospective cohort study

**DOI:** 10.1186/s40463-021-00544-8

**Published:** 2021-11-12

**Authors:** Ryan K. Chan, Beau Ahrens, Paul MacEachern, J. Douglas Bosch, Derrick R. Randall

**Affiliations:** 1 Cumming School of Medicine, University of Calgary, Calgary, AB Canada; 2 Interdisciplinary PhD Program, Dalhousie University, Halifax, NS Canada; 3 Interventional Pulmonary Medicine, Division of Respirology – Thoracic Surgery and Medical Oncology, Cumming School of Medicine, University of Calgary, Calgary, AB Canada; 4 Section of Otolaryngology – Head and Neck Surgery, Department of Surgery, Cumming School of Medicine, University of Calgary, Calgary, AB Canada; 5 Calgary Voice Clinic, Richmond Road Diagnostic and Treatment Centre, Calgary, AB Canada

**Keywords:** Subglottic stenosis, Idiopathic, Alberta

## Abstract

**Background:**

Subglottic stenosis (SGS) is a reportedly rare disease that causes recurrent severe airway obstruction. Etiologies reported for SGS include idiopathic, iatrogenic, autoimmune, congenital, and traumatic, with variable ratios among different centres. From empiric observation, southern and central Alberta was hypothesized to have a disproportionate distribution of SGS driven by increased idiopathic SGS (iSGS) compared to previous literature. Identification of causative agents of iSGS will help understand and guide future management options, so this study aimed to characterize the demographics of SGS subtypes, define prevalence and incidence rates of iSGS in southern Alberta, and geographically analyze for clustering of iSGS prevalence.

**Methods:**

SGS patients from Alberta census divisions No. 1–9 and 15 were retrospectively reviewed. Patients were subtyped according to etiology of SGS and characterized. Idiopathic SGS prevalence and incidence was assessed; prevalence was further geographically segregated by census division and forward sortation area (FSA). Significant clustering patterns were assessed for using a Global Moran’s I analysis.

**Results:**

From 2010 to 2019 we identified 250 SGS patients, who were substantially overrepresented by idiopathic patients (80.4%) compared to autoimmune (10.0%), iatrogenic (7.6%), congenital (1.2%), and traumatic (0.8%). The total iSGS prevalence was 9.28/100,000 with a mean annual incidence rate of 0.71/100,000 per year. Significant clustering was observed (Moran’s index 0.125; z-score 2.832; *p* = 0.0046) and the highest rates of prevalence were observed in southern Alberta and in rural communities heterogeneously dispersed around Calgary FSAs.

**Conclusion:**

In southern and central Alberta, iSGS patients were disproportionately over-represented in contrast to other subtypes with the highest prevalence in southern Alberta. There was a three-fold higher annual incidence compared to previous literature demonstrating the highest rates of disease reported worldwide. Future research aims to expand the geographical scope and to assess for demographic or environmental differences within significant clusters that may contribute to disease pathophysiology.

**Level of evidence:**

III.

**Graphical Abstract:**

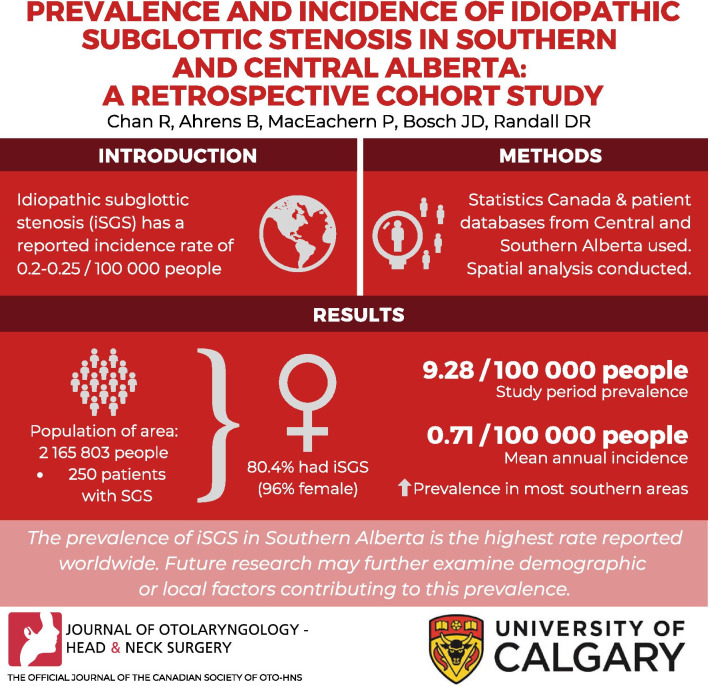

## Introduction

Subglottic stenosis (SGS) is the narrowing of the airway immediately below the vocal folds causing stridor and dyspnea that impairs functional capacity and quality of life [1]. Etiologies of SGS include idiopathic, iatrogenic, autoimmune, congenital, and traumatic, each with their own stenosis morphology, demographic patterns, degree of stenosis progression, and rates of recurrence [2, 3]. Different centres have reported varying ratios of prevalence among these subtypes with iatrogenic and idiopathic causes reported most commonly [2–6]. Idiopathic SGS (iSGS) is a challenging subtype of SGS to research and treat as it is reportedly rare but highly recurrent disease [7]. Although iSGS is a diagnosis of exclusion, it interestingly affects a homogenous population of primarily perimenopausal, Caucasian females [2, 8–10]. The specific etiology of iSGS remains unclear, however proposed pathophysiological factors include gastroesophageal reflux disease (GERD), upregulation of estrogen receptors in the subglottis, pathogenic bacterial flora, and epigenetic changes to inflammatory constituents of the subglottic mucosa [11–14].

The mainstay iSGS treatment involves repeated endoscopic incision and dilation techniques, along with combinations of adjuvant corticosteroids and mitomycin C; stenosis typically recurs within 1–3 years [10]. For refractory iSGS, cricotracheal resection (CTR) has been used to provide patients with long term symptomatic relief. However, CTR is a complex surgery with potential complications including dysphagia and dysphonia [8, 15–17]. In addition, a restenosis rate of 1.2–13% has been reported in iSGS patients following CTR with a mean restenosis latency from 4.3 to 12.5 years [8, 18]. Identification of factors contributing to iSGS development and propagation will be valuable in the long-term management of this debilitating condition.

Current literature suggests the population incidence of iSGS ranges from 0.2/100,000 to 0.25/100,000 person years in the Netherlands and United States, respectively [9, 19]. In Calgary, our tertiary centre provides care for all iSGS patients in southern and central Alberta, in addition to some patients living in neighbouring southeast British Columbia and southern Saskatchewan. Based on clinical observations and experience in our region, we hypothesized there is increased regional incidence of iSGS considerably greater than previous reports. This population-based retrospective review aimed to determine the distribution of SGS subtypes and their respective demographics, while determining the prevalence and incidence rates of iSGS within southern and central Alberta. Analysis for geographic clustering of disease was further investigated in hopes to provide additional insight into disease etiology.

## Methods

### Patient identification

This retrospective study was approved by the University of Calgary Conjoint Health Research Ethics Board (REB19-1076). Effectively all SGS patients in southern and central Alberta receive care through two primary programs: the Calgary Voice Clinic (CVC) and the southern Alberta Interventional Pulmonary Medicine Centre (IPMC), which are serviced by otolaryngologists and interventional pulmonologists. Subglottic stenosis patients from the CVC were identified from a prospectively collected clinic patient database, plus review of all operative dates and procedures from the otolaryngology surgeons’ (JDB, DRR) electronic medical record (EMR), to capture additional patients not seen at the CVC from January 1, 2010 to December 31, 2019. Patients from the IPMC were identified through the Stather Canadian Outcomes registry for chest ProcedurEs (SCOPE) Synoptec operative summaries database detailing patients who underwent a diagnostic or therapeutic bronchoscopy between 2017–2019 [20]. Identified patients were cross-referenced to remove duplicates.

Subglottic stenosis was defined as narrowing of the subglottis within 2 cm below the vocal cords as visualized by laryngoscopy or bronchoscopy). Patients with SGS extension superiorly to the glottis and/or inferiorly to involve proximal trachea were included. Patients with no documented SGS (including distal tracheal stenosis, neoplastic subglottic masses, and bilateral vocal fold fixation without SGS) and pediatric patients (< 18 years of age at time of data collection) were excluded. Southern and central Alberta were defined as Statistics Canada 2016 census divisions No. 1–9 and 15 of Alberta [21]. The CVC and IPMC primarily services southern and central Alberta while Edmonton provides care to the majority of patients north of these census divisions. Therefore, patients with a primary residence from another province or from northern Alberta census divisions were excluded from data analysis to provide the most accurate incidence and prevalence values for this region.

**Fig. 1:**
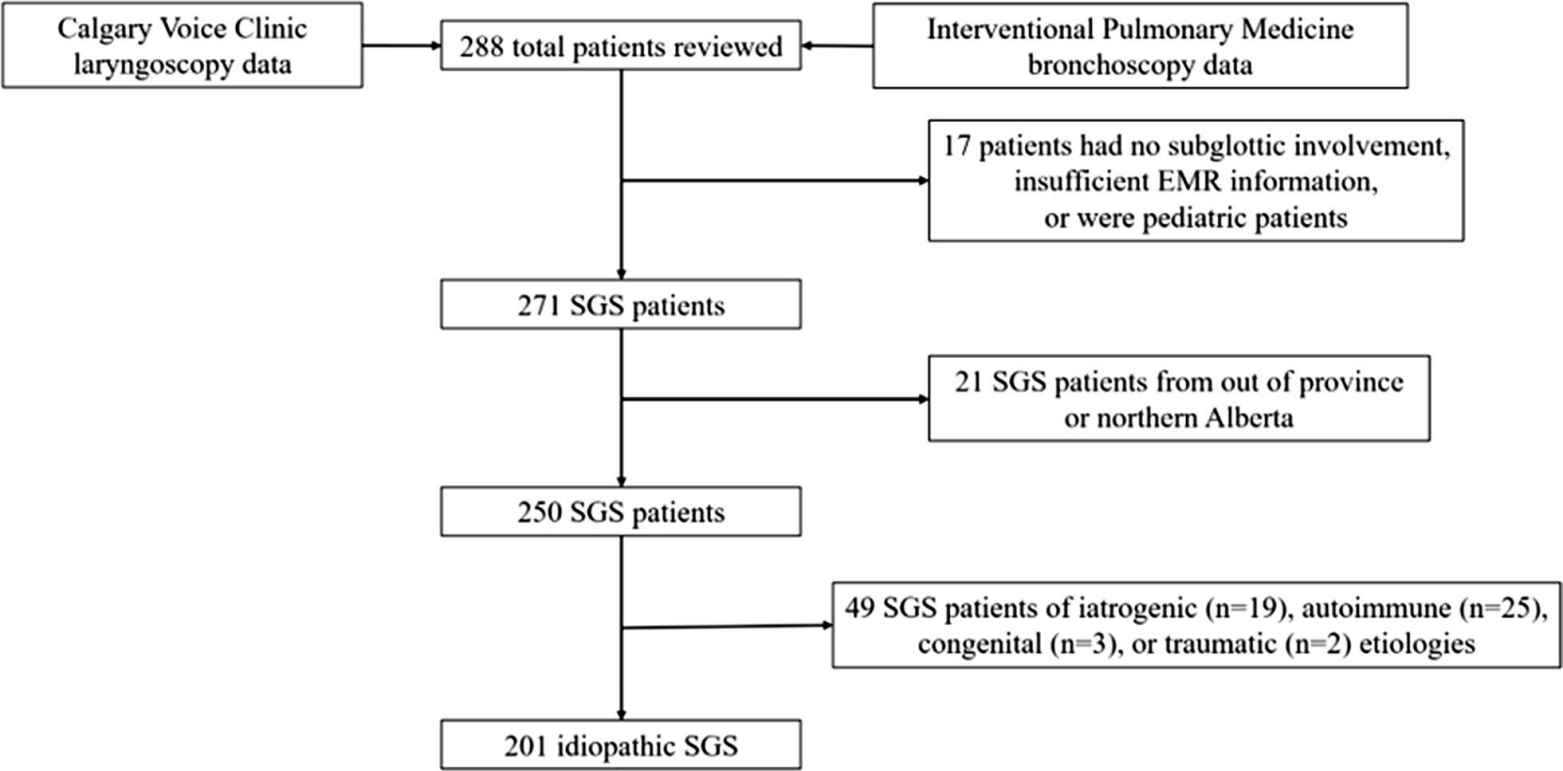
Consort diagram displaying patient exclusion criteria. Patients were identified using Calgary Voice Clinic laryngoscopy data and Interventional Pulmonary Medicine bronchoscopy data. Patients were excluded if they had no subglottic involvement, insufficient information in the EMR, was a pediatric patient, or from out of province/northern Alberta. Patients were then classified into idiopathic, iatrogenic, autoimmune, congenital, or traumatic subtypes with breakdown of their distribution noted. Idiopathic SGS were the primary subtype of interest and further analysis performed regarding prevalence and incidence geographic distribution. EMR = electronic medical record, SGS = subglottic stenosis

Patients were classified into subtypes according to criteria used in comparable literature [2]. Idiopathic SGS was defined as the exclusion of established causes associated with SGS: no history of laryngotracheal injury, caustic or heat burn, rheumatologic markers, signs of systemic autoimmune disease, nor major neck surgery involving the trachea within two years prior to symptom onset. If symptoms developed within two years following intubation, tracheostomy, or airway surgery then patients were considered iatrogenic SGS. Documented clinical history including serologic or histologic diagnosis of known autoimmune conditions associated with SGS, including granulomatosis polyangiitis (GPA), rheumatoid arthritis (RA), IgG4-disease, systemic lupus erythematosus (SLE), and sarcoidosis classified patients as autoimmune SGS. Patients were recorded as traumatic if symptom onset was within two years following external or internal laryngotracheal trauma, including ingestion or inhalation of heat or caustic substances. Lastly, SGS was classified as congenital if there was history of SGS symptoms in first five years of life with no identified etiologies as above. Individual patient demographics, primary residence postal code, indications of disease etiology, and comorbidities were collected from EMRs. Comorbidities were utilized to calculate a Charlson-Comorbidity Index (CCI) score. Exclusion criteria along with SGS subtype segregation are displayed in Fig. 1.

### Statistical analysis

Prevalence and incidence were calculated based on population information collected from the Statistics Canada 2016 Canadian Census, which was the most chronologically suitable for this study period [21]. Incidence was calculated based on the number of new cases diagnosed *per annum* from January 1, 2010, to December 31, 2019. This time period reflected the introduction of the clinic EMR to ensure comprehensive patient identification. Statistical analyses were done using IBM SPSS Statistics version 26.0.0 (IBM Corp, Armonk, NY). Univariate analyses of patient characteristics were performed using one-way analysis of variance (ANOVA) with Games-Howell *post-hoc* comparisons, χ^2^ test, or Fisher’s exact test where appropriate. Threshold for statistical significance was *p* < 0.05. Correlation of annual incidence rate over years was performed using the Pearson coefficient with a statistical significance threshold of *p* < 0.05.

### Spatial analysis

To analyze spatial trends of iSGS prevalence distribution, patients were stratified first by census divisions and then by Forward Sortation Areas (FSA). FSAs are the first three digits of the six digit Canadian postal code based on reported residence as well as a Statistics Canada supported administrative boundary unit. Six digit postal code boundaries are aligned with FSAs, thereby facilitating precise and accurate aggregation of postal codes to this larger spatial unit. The 2016 census year FSA boundaries were obtained from Statistics Canada for all of Alberta [21]. All spatial analyses were performed using ArcGIS Pro® 2.6 (ESRI, Toronto, ON). FSAs corresponding to southern and central Alberta were isolated and all patient records were geocoded to respective FSAs.

Once all cases were geocoded, 2016 Canadian Census population numbers were likewise coded by FSA using Statistics Canada tabular datasets. Prevalence rates of iSGS (cases per 100,000) were then calculated for each FSA. Using prevalence rather than raw incident rates allowed for standardization and ensured validity of statistical analyses. To determine whether there is spatially significant autocorrelation, a Global Moran’s I analysis was performed. This analysis evaluated whether the spatial distribution of iSGS exhibited a significant pattern of clustered (positive value) or dispersed (negative value) cases in comparison to a random pattern.

## Results

### Subglottic stenosis subtypes and patient characteristics

A total of 288 patients were identified and a cohort of 250 patients with SGS from southern and central Alberta was identified (Fig. 1). Presenting symptoms were primarily progressive dyspnea, stridor, and chronic cough, with very few asymptomatic patients. Demographic and comorbidity characteristics based on SGS subtype are shown in Table 1. When stratified by subtype, 201 iSGS patients encompassed the majority of cases (80.4%) with small contributions from autoimmune (n = 25, 10%), iatrogenic (n = 19, 7.6%), congenital (n = 2, 1.2%), or traumatic (n = 2, 0.8%) causes. Since congenital and traumatic subtypes represented only five cases, they were removed from further demographic and comorbidity statistical comparisons. In our series, nine cases reported a positive family history involving a first-degree family member. Idiopathic SGS was nearly exclusively female (n = 192, 96%) and diagnosed within a similar age range (47.77 ± 12.29 years) to autoimmune and iatrogenic SGS. Autoimmune SGS (n = 17, 68%) showed less female preponderance compared to iatrogenic (n = 17, 89%) and idiopathic (n = 192, 96%; Table 1, *p* < 0.001).

**Table 1:** Demographics and comorbidities grouped by subglottic stenosis subtype (total sample included 250 patients of all etiologies). Idiopathic patients showed similar features to iatrogenic and autoimmune patients, though some differences were observed among rheumatologic, lung, and liver diseases

	Idiopathic	Iatrogenic	Autoimmune	*p* value
*Demographics*				
Number, n (%)	201 (80.4%)	19 (7.6%)	25 (10%)	
Age, years (mean ± SD)	54.6 ± 13.48	53.8 ± 21.93	49.3 ± 16.8	0.246
Age at diagnosis, years (mean ± SD)	47.8 ± 13.3	48.8 ± 22.9	43.0 ± 16.3	0.276
Sex, n (% female)	192 (95%)	17 (89%)	17 (68%)	< 0.001*
*Comorbidities*				
Number of comorbidities (mean ± SD)	1.60 ± 1.35	3.37 ± 2.11	3.04 ± 2.35	< 0.001*
Charlson comorbidity index (mean ± SD)	0.25 ± 0.86	0.79 ± 1.03	1.04 ± 0.54	< 0.001*
Age-adjusted Charlson comorbidity index, (mean ± SD)	1.44 ± 1.62	2.21 ± 2.10	1.96 ± 1.46	0.063
Gastrointestinal				
Gastroesophageal reflux disease, n (%)	65 (32%)	6 (32%)	9 (36%)	0.964
Peptic ulcer disease, n (%)	0	1 (5%)	1 (4%)	0.084
Inflammatory bowel diseases, n (%)	16 (8%)	3 (16%)	1 (4%)	0.346
Liver disease, n (%)	0	2 (11%)	1 (4%)	0.003*
Metabolic/endocrine				
Hypothyroidism, n (%)	25 (12%)	2 (11%)	2 (8%)	0.926
Diabetes, n (%)	15 (7%)	5 (26%)	1 (4%)	0.028*
Dyslipidemia, n (%)	18 (9%)	2 (11%)	2 (8%)	0.909
Cardiac				
Hypertension, n (%)	42 (21%)	8 (42%)	6 (24%)	0.106
Myocardial infarction, n (%)	4 (2%)	1 (5%)	0	0.398
Coronary artery disease, n (%)	2 (1%)	1 (5%)	0	0.242
Congestive heart failure, n (%)	2 (1%)	1 (5%)	0	0.242
Stroke, n (%)	2(1%)	0	0	1.000
Neurologic/psychiatric				
Fibromyalgia, n (%)	6 (3%)	1 (5%)	3 (12%)	0.072
Migraines, n (%)	5	5	0	0.691
Other psychiatric illnesses, n (%)	10	32	12	0.061
Autoimmune/rheumatologic				
Granulomatosis with polyangiitis, n (%)	0	0	14 (56%)	< 0.001*
Rheumatoid arthritis, n (%)	0	1 (5%)	7 (28%)	< 0.001*
Other connective tissue disorders, n (%)	0	0	3 (12%)	0.001*
Respiratory				
Chronic obstructive pulmonary disease, n (%)	2 (1%)	2 (11%)	0	0.043*
Asthma, n (%)	9 (4%)	5 (26%)	3 (12%)	0.002*
Renal				
Chronic kidney disease, n (%)	3 (2%)	0	0	1.000

*SD* standard deviation

*p < 0.05.

There was a significant difference among the subtypes in the CCI (*p* < 0.001), but not when this was age adjusted (*p* = 0.133). Table 1 shows individual comorbidities among the different subtypes, with iSGS and autoimmune patients reporting lower proportions of asthma (*p* = 0.002), liver diseases (*p* = 0.003), diabetes mellitus types 1 or 2 (*p* = 0.028), and chronic obstructive pulmonary disease (COPD) (*p* = 0.043) compared to iatrogenic SGS patients. There was no significant difference in the percentage of patients with GERD among the SGS subtypes (*p* = 0.964).

### Prevalence and incidence rates of idiopathic SGS

**Fig. 2:**
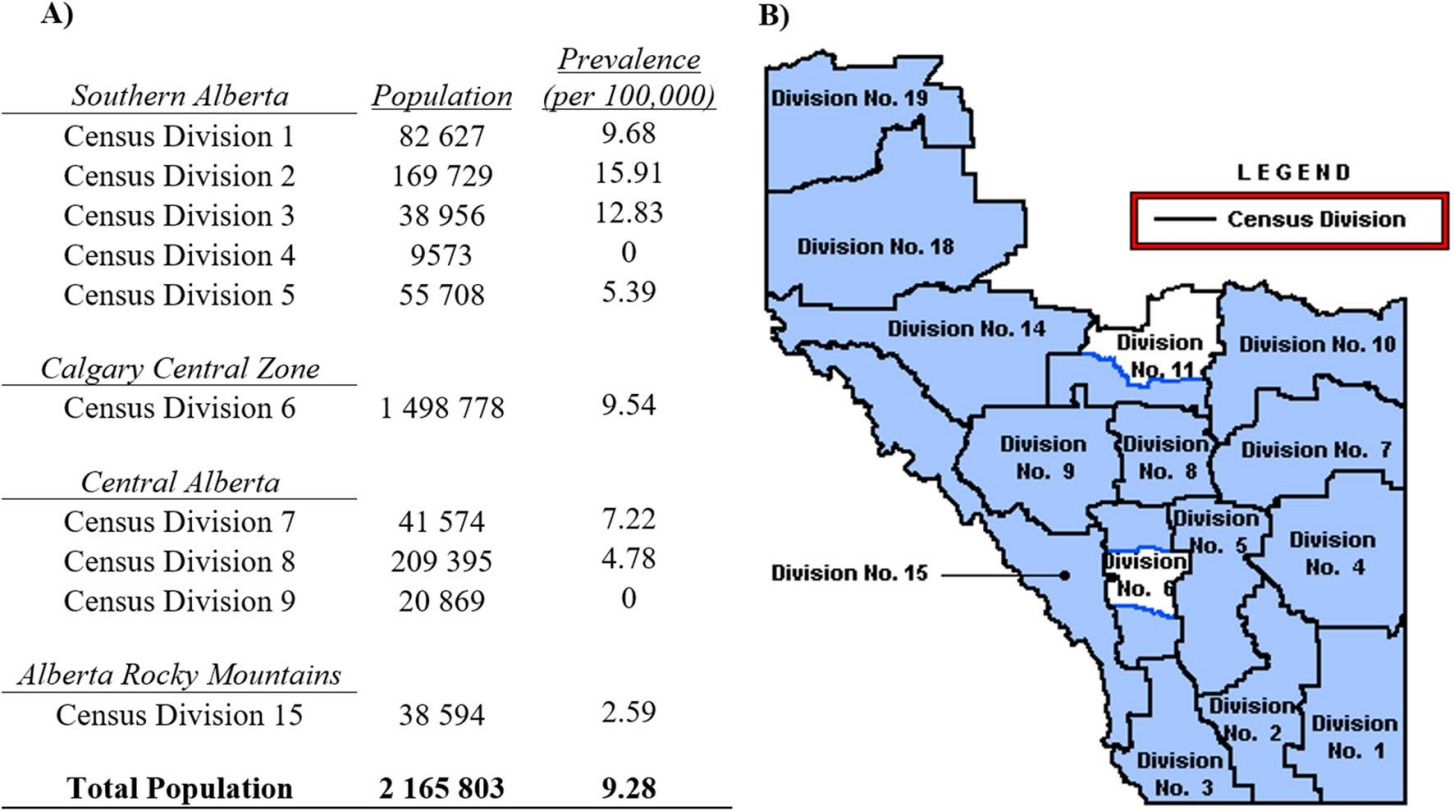
Prevalence of idiopathic subglottic stenosis in southern and central Alberta from 2010–2019 based on the Statistics Canada 2016 Census. **A** Total population of southern and central Alberta as defined by the census divisions No. 1–9 and 15, and the calculated prevalence of idiopathic subglottic stenosis per 100,000. **B** Alberta map segregated by census divisions [43]

**Fig. 3:**
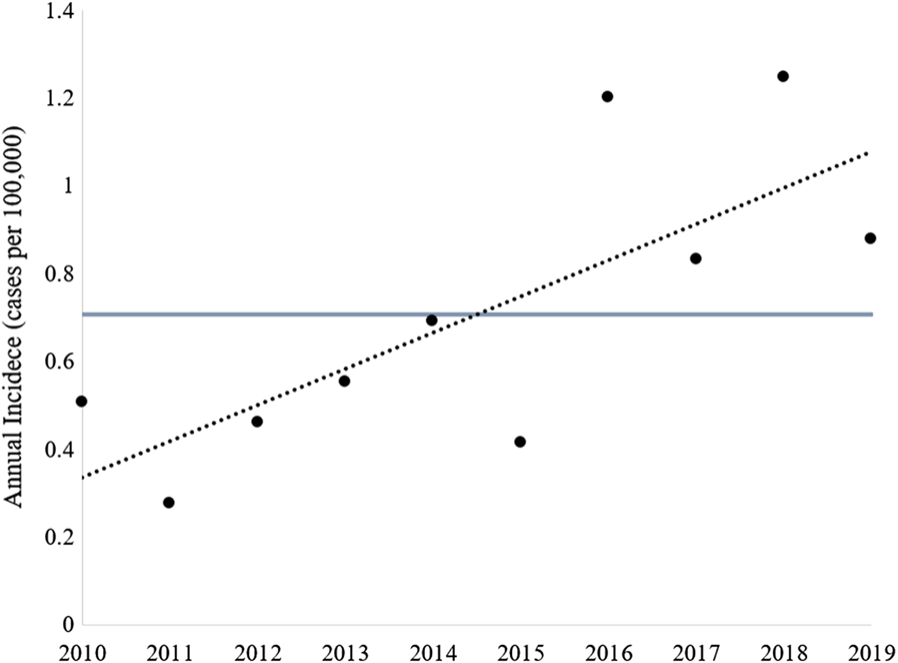
Scatter plot representing annual idiopathic subglottic stenosis incidence based on new diagnosis over 2010–2019. Blue line represents the mean annual incidence rate at 0.71/100,000 per year. Dotted line represents the trend line with a significant correlation of r^2^ = 0.578, *p* = 0.011

Prevalence and incidence rates of iSGS in southern and central Alberta were derived from a total population of 2,165,803 based on the cumulative population among census divisions No. 1–9 and 15 (Fig. 2). During the study period, 201 iSGS patients received care through the CVC and IPMC leading to a period prevalence of 9.28/100,000 persons. The mean annual incidence during the past 10 years was 0.71/100,000 persons per year (Fig. 3). There was a significant positive correlation with annual incidence rate of iSGS increasing over the 10 year period (r^2^ = 0.578, *p* = 0.011; Fig. 3).

### Spatial trends of idiopathic SGS prevalence

The iSGS prevalence rate of southern and central Alberta was further geographically analyzed by census division and FSA. There was a general trend of increasing iSGS prevalence moving from central to southern census divisions (Fig. 2), with the highest prevalence areas in the three most southern census divisions: No. 2 (15.91/100,000), No. 3 (12.84/100,000), and No. 1 (9.68/100,000). Census divisions in No. 4 and No. 9 had no iSGS cases from 2010 to 2019.

**Fig. 4:**
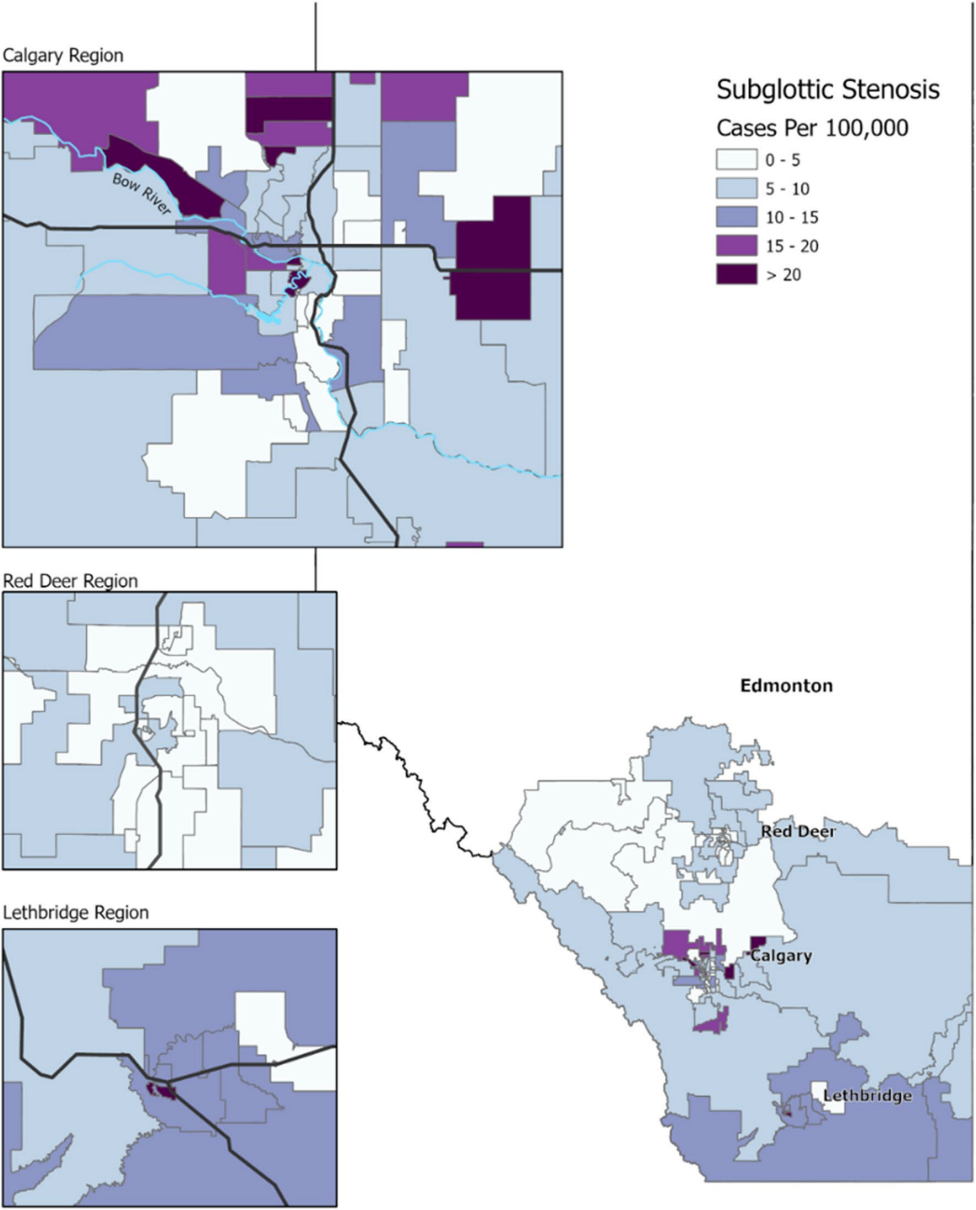
Prevalence map of idiopathic subglottic stenosis patients in southern and central Alberta stratified by FSA region and normalized by the respective FSA population in cases per 100,000. FSA = forward sortation area

A total of 199 from 201 input cases of iSGS were successfully geocoded to the study area FSAs (Fig. 4). The aggregated cases by FSA range from 0 to 30.64 cases per 100,000 people with a mean period prevalence by FSA of 8.58/100,000. Again, the same of trend of increasing iSGS prevalence in southern Alberta was observed. There was evident variability in iSGS prevalence of FSAs within and surrounding Calgary. Through a Global Moran’s I analysis, we found a significant positive spatial autocorrelation (clustered distribution pattern) of iSGS cases in southern and central Alberta (Moran’s index 0.125; z-score 2.832; *p* = 0.0046). Therefore, there was less than a 5% chance that clustering patterns could be the result of random chance.

## Discussion

Our study confirmed the clinical impression that iSGS was present at a higher prevalence and incidence within southern and central Alberta than would be expected based on existing literature. From the 250 adult SGS patients identified, iSGS formed the overwhelming majority of cases and represented an appreciable burden of disease unique to this region. Despite the elevated number of iSGS patients observed, the demographic characteristics still reflected the typical profile—almost exclusively perimenopausal females [2, 8–10]. This indicated that the abnormally high rate of disease was most likely an intrinsic factor to the population, such as genetic makeup or socioeconomic elements, possibly in combination with environmental agents.

When compared to studies from the United States, the Netherlands, and Australia, the most remarkable differences in this investigation were the disproportionately high number of SGS patients skewed towards the idiopathic subtype (80.4%). Firstly, this study contained the largest number of SGS patients (n = 250) recruited from by a single urban centre even after excluding 21 SGS patients residing outside the defined area of southern and central Alberta [2–6]. In contrast to our findings, the US and Netherlands studies reported the predominant SGS subtype as iatrogenic with idiopathic representing only 16.8–18.5% and 20%, respectively [2, 3, 6]. Subsequent investigations revealed closer iSGS distributions of 45% in Los Angeles, California and 53% in Victoria, Australia, but no previous studies show the degree of skew towards iSGS we observed in Alberta [4, 22]. While ethnicity was not specifically evaluated in this study and not collected in EMRs in our population, the province of Alberta has approximately one quarter of its population self-identify as a visible minority [23]. Calgary is Alberta’s largest city and has the highest proportion of residents identify as visible minorities (33.7%), so we expect this population would be comparable to other urban centres, albeit with a lower proportion of Caucasian population with respect to Groningen, Netherlands, but slightly higher than Little Rock, AR; Houston, TX; Los Angeles, CA; and Victoria, Australia [23–28].

Our SGS classification criteria was designed to match previous reports, but minor differences in inclusion and exclusion criteria among the studies, such as how previous intubation timelines and comorbidities are addressed, may partially account for this variation in SGS stratification. However, we postulate there are yet undetermined regional factors specific to southern and central Alberta resulting in the higher proportion of iSGS patients. Relationship to intubation will always challenge accurate classification. Our lower observed proportion of iatrogenic SGS patients may indicate bias owing to classification overlap; some patients with an underlying idiopathic SGS may develop upper airway obstruction symptoms within two years of intubation that may not have been necessarily traumatic, or the intubation may have triggered a stenosis in a patient predisposed to reactive inflammation and stenosis development. Iatrogenic patients also had the highest CCI that was reflected in the higher proportions of asthma, liver disease, diabetes, and COPD, which may increase a patient’s likelihood to require intubation (Table 1). Furthermore, prior studies of iSGS reported higher prevalence of GERD compared to the general population rate (approximately 15% in North America) [29–34]. Although GERD was also present at higher than population rates among our iSGS population (32%), iatrogenic and autoimmune subtypes also shared similarly higher rates of GERD. However, the presence of GERD was taken from patient histories and frequently reflected a clinical diagnosis from primary care or other practitioners; only a limited number of patients underwent reflux testing or endoscopy.

The 2010–2019 iSGS period prevalence at our tertiary centre was found to be 9.28/100,000 persons with a mean annual incidence rate of 0.71/100,000 persons per year (Fig. 2). There were no previously reported prevalence rates of iSGS available for comparison, while annual incidences of iSGS in the US and the Norway have been estimated to be 0.25/100,000 and 0.20/100,000, respectively [9, 19]. In comparison, our results revealed approximately three-fold higher rate of new diagnoses annually. A major strength of our health care system is the centralization of tertiary services, including airway procedures, and a single health care governing body that manages all clinical encounters across the province. Therefore, our incidence and prevalence rates accurately reflect the entire region, whereas comparable reports of iSGS incidence rates were from single-centre studies and may not represent the true incidence rate within their health regions. Our investigation determined incidence based on new diagnoses of iSGS per year within a well-defined region and time frame, which provides the most accurate incidence rate; previous studies included events of disease recurrence to calculate incidence rate or identified diagnoses over a time period rather than specific years [9, 19]. Lastly, when the annual incidence rate of iSGS was measured over time, there was a steady increase in annual incidence in recent years (Fig. 3). One factor that may impact this annual incidence trend is the IPMC database identified patients undergoing bronchoscopies between 2017 and 2019. Therefore, if previously diagnosed IPMC iSGS patients did not require a procedure during this three year period, they were unaccounted for and may underestimate the total prevalence and incidence rate of earlier years. Existing literature indicates that 50% of iSGS patients have recurrence of stenosis requiring treatment within 1.5 years and 80% by approximately 3 years [10]. Therefore, we expect a minimal number of potential patients unaccounted for with the exception of those who died or left the region prior to 2017.

Assessing prevalence of iSGS within specific geographic regions of southern and central Alberta at increasing levels of granularity provides the opportunity to understand how disease is distributed. The iSGS prevalence of each specific village/town/city was not performed because of the extreme discrepancy of total population when comparing villages to large urban centers. Many iSGS patients were from rural communities, some of which had populations less than 100 people. Therefore, aggregation into FSAs provided a better representation of the geographic distribution of iSGS prevalence. One may assume increased prevalence of disease in regions closest to tertiary care due to increased access to specialty healthcare, as seen with some clusters near Calgary; however, the highest prevalence of iSGS was observed in the most southern census divisions and FSAs (Figs. 2 and 4), which strongly supports an agent involved in iSGS development in those regions. There may be some patient attrition from central Alberta to Edmonton, as the other tertiary centre in Alberta, affecting the relative prevalence of more central FSAs. Calgary provided care for five patients from northern Alberta (in addition to sixteen patients from British Columbia and Saskatchewan) who were excluded in this analysis, so the converse may be true simply as a result of referral patterns.

An interesting observation was the geographic heterogeneity within Calgary and the surrounding FSAs. The Global Moran’s I analysis revealed significant positive geospatial autocorrelation, indicating clustering of increased iSGS prevalence FSAs was unlikely attributable to random chance. Clustering occurs when there are the groupings of high prevalence FSAs together, or vice versa where low prevalence FSAs are grouped together. This analysis factors in all FSAs as a whole to examine for significant clustering patterns within a specified general area, but it does not reveal where these clusters are located. Within Calgary, FSAs with greater than 15 cases per 100,000 were detected in the west and northwest regions. In the regions neighbouring Calgary, FSAs with greater than 15 cases per 100,000 were found in nearby rural communities north, east, and south of Calgary. One may postulate that these highlighted FSAs have higher iSGS prevalence due to the demographic homogeneity of the iSGS subtype. Calgary’s west and northwest FSAs and the surrounding rural communities may have a higher demographic proportion of perimenopausal Caucasian females compared to other regions due to socioeconomic factors and western European heritage of many rural agricultural communities. Further research focusing on analyzing statistically significant spatial outliers of high or low disease prevalence can be enhanced by incorporation of Statistics Canada census data. This would enable future comparisons of demographic, socioeconomic, and environmental factors associated with these FSAs to provide more insight into disease etiology.

Though SGS is a rare disease, there is growing interest in the underlying pathophysiology with investigations connecting pathogenesis of iSGS to a variety of agents, including cytokine and inflammatory cell dysfunction, some of which are implicated in autoimmune and inflammatory diseases and mycobacterial infection [13, 35–40]. This evolving understanding of iSGS pathogenesis provides testable hypotheses for new investigations regarding local environmental factors potentially explaining the increased prevalence and incidence rates of iSGS in southern and central Alberta. Moreover, hereditary factors may contribute to the observed high case burden of iSGS in this region, as described in previous data reporting multiple independent families with more than one member affected by iSGS in this region [41]. However, the majority of cases in our sample appear sporadic, so this warrants further investigation.

Additional limitations of this paper relate to its retrospective nature, classification of SGS, and chronology of residency. Despite attempts to standardize SGS classification criteria to previous literature, retrospective reviews are inherently subjective to classification bias. This study included SGS patients that had extension superiorly into the glottis and inferiorly to the distal trachea. It is possible this study risks misclassifying patients with glottic stenosis extending into the subglottis; however, there was a very limited number of posterior glottic stenosis patients not related to intubation or surgical trauma, so our exclusion criteria minimize this risk. Additionally, no formal grading scale of stenosis was used and SGS was determined based on direct visualization of mucosal narrowing within 2 cm inferior to the vocal cords, and therefore may be subjective to observer bias. While this study aimed to identify the incidence and prevalence of stenosis, incorporating grading and delving further into those patients with symptomatic disease will be pertinent in future studies. Lastly, prevalence was based on current location of residency rather than location of residency at symptom onset or age of diagnosis, and additionally does not consider the duration lived in that of other communities. Therefore, any potential associations regarding location residency and disease etiology would be confounded by this limitation.

Future investigations will be aimed at expanding patient recruitment and increasing resolution regarding location of residency. Our data cannot state, without data describing the Edmonton iSGS population, whether our high iSGS prevalence is unique to southern Alberta, a provincial phenomenon, or part of a larger epidemiologic focus. Including other Canadian centres treating iSGS may further identify distribution, prevalence, and incidence of SGS across the country to confirm or refute the increased disease rates in Alberta. Efforts from the North American Airway Collaborative (NoAAC) Network that include the US, Australia, and United Kingdom have been effective in creating an online social networking platform for patient education, patient peer support, and research [8, 42]. Similarly, establishing a Canadian network and promoting collaboration would greatly enhance research into this rare but perplexing disease. Comparisons among centres may allow us to identify these differences and explore why they exist to optimize care for iSGS patients.

## Conclusion

Southern and central Alberta has the highest proportion of iSGS patients based on prevalence and incidence of this rare disease reported in the world literature. Geospatial analysis revealed clusters of high prevalence FSAs that have a heterogenous distribution. Though the mechanism behind iSGS pathogenesis is unknown, this study identifies an enriched population to support future investigations aiming to identify demographic differences across significant clusters of prevalence, and to investigate for local factors that may explain the increased prevalence and incidence of iSGS.

## Data Availability

The datasets used and/or analysed during the current study are available from the corresponding author on reasonable request.

## Abbreviations

ANOVA: Analysis of variance
CCI: Charlson comorbidity index
COPD: Chronic obstructive pulmonary disease
CVC: Calgary Voice Clinic
EMR: Electronic medical record
FSA: Forward sortation area
GERD: Gastroesophageal reflux disease
GPA: Granulomatosis polyangiitis
IPMC: Interventional Pulmonary Medicine Clinic
iSGS: Idiopathic subglottic stenosis
*MtbC*: Mycobacterium tuberculosis complex
RA: Rheumatoid arthritis
SCOPE: Stather Canadian Outcomes registry for chest ProcdurEs
SGS: Subglottic stenosis
SLE: Systemic lupus erythematosus
